# Three metachronous primary lung cancers in a chronic smoker: A case report and review of the literature

**DOI:** 10.1097/MD.0000000000022559

**Published:** 2020-12-18

**Authors:** Dushyant Damania, Lillian Chow, Boris Betancourt, James Mahoney, M.A. Haseeb, Absia Jabbar, Raavi Gupta, Gurinder Sidhu

**Affiliations:** a Division of Pulmonary and Critical Care, Department of Medicine; b Department of Pathology; c Division of Hematology and Oncology, Department of Medicine, State University of New York Downstate Health Sciences University, Brooklyn, New York.

**Keywords:** metachronous lung cancer, small cell lung cancer, smoking, smoking cessation

## Abstract

**Rationale:**

Lung cancer is a leading cause of cancer-related deaths. Smoking is major risk factor for initial and subsequent lung cancer especially in active smokers. Treatment of subsequent lung cancer depends on whether it is synchronous or metachronous. We report a rare case of triple metachronous lung cancer and review of literature of patients with triple metachronous cancers. This will be the second case reported of triple metachronous lung cancer.

**Patient concerns:**

A 60-year-old male, active smoker with diabetes mellitus, chronic obstructive pulmonary disease (COPD) and peripheral arterial disease presented with cough and hemoptysis. Initial computed tomography (CT) scan showed right upper lobe spiculated mass.

**Diagnosis:**

He underwent transthoracic needle biopsy for right upper lobe mass, showing primary lung adenocarcinoma (ADC)-Stage-IIIA. He continued to smoke and 9-years later had new left upper lobe spiculated nodule, which on surgical resection showed squamous cell carcinoma (SCC)-Stage-IA1. Despite counselling on smoking cessation, he was unable to quit. Six months later, he presented with shortness of breath and CT chest showing right hilar adenopathy in right upper and lower lobes. He underwent transbronchial biopsies of lesion which showed small cell lung carcinoma (SCLC).

**Interventions:**

His initial lung ADC-Stage-IIIA, was treated with chemotherapy, weekly thoracic radiation and additional chemotherapy cycles. Nine years later, his left upper lobe mass showing SCC-Stage-IA1 was deemed curative after apical resection and he was kept on surveillance. Six months later, after diagnosis of SCLC in right upper and lower lobe, patient was not a candidate for systemic chemotherapy due to poor performance status and opted for hospice care.

**Outcomes:**

His initial lung ADC-Stage-IIIA showed complete radiological response with chemotherapy and radiation. Subsequent SCC-Stage-IA1 was deemed curative after resection. Due to his poor performance status, he was not a candidate for chemotherapy for SCLC and patient opted for hospice care.

**Lessons:**

Smoking is a major risk factor for developing lung cancer and with continued smoking, patients are at higher risk for developing subsequent primary lung cancers. We recommend, patients with lung cancer must quit smoking, and those who do not, should remain on long-term surveillance.

## 1 Introduction

Lung cancer is the most common cause of cancer-related death in the United States where an estimated 234,000 new cases were diagnosed in 2018.^[1]^ After definitive treatment, surveillance with computerized tomography (CT) scan is recommended for 5 years or longer. The appearance of lung nodule(s) or mass (es) in a patient treated previously for lung cancer presents a diagnostic challenge. Malignant masses in such patients could represent a second primary lung cancer or recurrent and/or metastatic disease. These masses could present simultaneously at first diagnosis of cancer (synchronous) or subsequent to initial diagnosis (metachronous). The differentiation of these neoplasms poses a challenge toward staging, management, and prognosis. After curative resection of early stage disease, patients can develop a second primary lung cancer at a rate of **−**1.5% per patient year, however, the risk is likely higher in patients who continue to smoke.^[2]^ A metachronous second primary lung cancer is defined as a new tumor that develops after a cancer-free interval of ≥4 years or is of a different histological type. Recurrent cancer of the original histologic type diagnosed within 2 years of the initial diagnosis is usually a metastasis.^[2]^

Since metachronous lung cancers are so rare, we describe a case of an active long-term smoker who was diagnosed with lung ADC, treated definitively, developed a second primary lung cancer 9 years later, and then a third primary lung cancer within the same year. Each of the 3 tumors were of a different type. We have also compiled and reviewed clinical and pathologic features of other cases of synchronous and metachronous lung cancer from the literature.

## 2 Case description

**Figure 1:**
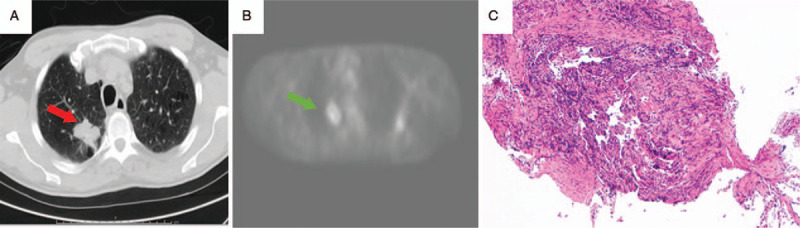
Radiologic imaging and histopathology of first primary lung carcinoma. A) CT scan of the chest showing right upper lobe. Note the 3.3 x 2.4 cm spiculated lung mass with stippled calcifications (red arrow). B) Non-fused PET scan of chest showing hypermetabolic lung mass (green arrow). C) Poorly differentiated adenocarcinoma (H&E, 100x).

This is a case of a 60-year-old male smoker (55 pack-year history) who presented with cough and hemoptysis. The patients wife provided informed consent for publication of the case. He had previously been diagnosed with diabetes mellitus, chronic obstructive pulmonary disease (COPD) and peripheral arterial disease but denied a family history of malignancy. Pulmonary function testing revealed moderately severe COPD without any restriction or gas transfer defect (Table 1). His oxygen saturation was >92% while breathing ambient air. His physical exam was only remarkable for clubbing and right basilar crackles. A computerized tomographic (CT) scan revealed a right upper lobe spiculated mass (3.3 x 2.4 cm) (Fig. 1A). Positron emission tomography (PET) scan showed 5 cm right upper lobe mass with standard uptake value (SUV) of 4.5 in the primary tumor (Fig. 1B) and ipsilateral hilar and paratracheal nodes in metastatic range. Fiberoptic bronchoscopy showed no endobronchial lesions, but histopathologic examination of a transthoracic needle biopsy showed poorly differentiated adenocarcinoma of the lung which was positive for TTF-1 (Fig. 1C). Bronchial washings were also positive for malignant cells. The cancer was staged to be Stage IIIA. Patient was treated with chemotherapy and radiation using paclitaxel 50 mg/m^2^ and carboplatin AUC 2 weekly with concurrent thoracic radiation. This was followed by 2 additional cycles of paclitaxel 200 mg/m^2^ and carboplatin AUC 6 every 21 days. Total dose of radiation delivered to the primary tumor, mediastinal, and hilar lymph nodes was 6660 cGy. CT scans at 3 months of completion of chemo- and radiotherapy showed that he had a complete radiologic response and was placed on surveillance. No recurrence was seen on periodic CT scanning, and the patient continued to be an active smoker, despite smoking cessation counselling.

**Table 1:** Results of initial pulmonary function tests at presentation.

	Patient data	Interpretation (% of reference)
FVC (L)	3.24	98
FEV1 (L)	1.28	50
FEV1/FVC (%)	40	50
TLC (L)	7.86	125
RV (L)	5.30	214
RV/TLC (%)	67	166
DLCO (ml/mmHg/min)	16.6	>80

**Figure 2:**
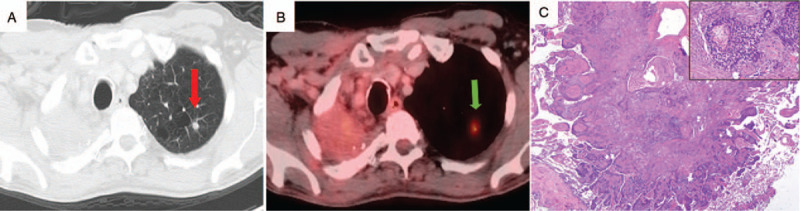
Radiologic imaging and histopathology of second primary lung carcinoma diagnosed 9 years after the first lung cancer diagnosis. A) CT scan of the chest showing 0.9 cm left upper lobe lung nodule (red arrow). B) PET/CT scan showing hypermetabolic left upper lobe lung nodule (green arrow). C) Moderately differentiated keratinizing type squamous cell carcinoma (H&E, 20x). Inset shows high-power view of a nest of malignant squamous cells (200x).

Nine years after initial cancer diagnosis, chest X-ray and then CT scan chest confirmed a new 9 x 5 mm posterior left upper lobe spiculated nodule (Fig. 2A). PET/CT showed the left upper lobe lung lesion to have an SUV of 4 (Fig. 2B). No new or enlarged mediastinal lymph nodes were identified and there was stable right sided post-radiation fibrosis. His laboratory investigations then were significant for polycythemia with hemoglobin of 18 gm/dl (reference range: 14–16 gm/dl), with normal white blood cells and platelets. The erythropoietin level was 22 IU/L (reference range: 2–18 IU/L) suggesting secondary polycythemia. Patient underwent video-assisted thoracoscopic surgery and apical wedge resection of the left upper lobe. Histopathological examination of the resected tumor showed moderately differentiated keratinizing squamous cell carcinoma (0.9 cm, stage IA1), with adjacent bronchioles showing squamous metaplasia (Fig. 2C). Molecular and cytogenetic studies were negative for ALK rearrangement and BRAF, EGFR, and KRAS mutations. PDL-1 expression was low [10% Tumor Proportion Score; antibody clone 22C3 (PharmDx)]. After discussion in multidisciplinary tumor board, it was decided not to offer adjuvant chemotherapy but to keep the patient on surveillance. He was given extensive counselling on smoking cessation, but he was still not able to quit.

**Figure 3:**
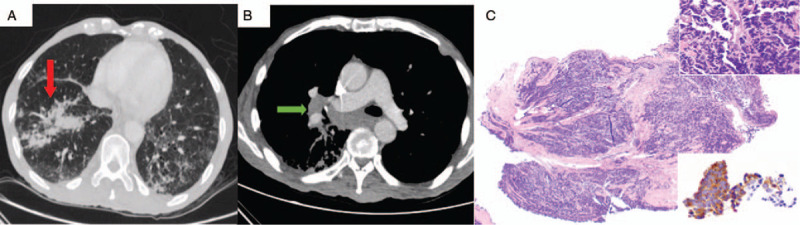
Radiologic imaging and histopathology of third primary lung cancer. A) CT scan of the chest showing perilymphatic nodules in the right lower lobe of the lung (red arrow). B) CT scan of the chest showing increased right hilar and mediastinal lymphadenopathy (green arrow). C) Small cell carcinoma (H&E, 40x). Upper inset shows high-power view of malignant cells with apoptosis. Lower inset shows positive synaptophysin expression in cancer cells (400x).

Six months later, patient presented with shortness of breath and CT of the chest showed increased soft tissue density and adenopathy of the right hilum (Fig. 3B) with perilymphatic nodules in the right upper and lower lobes (Fig. 3A) consistent with lymphangitic carcinomatosis concerning for recurrence. Patient was admitted to the hospital for unrelated left leg pain due to peripheral artery disease. Due to worsening disease, he developed gangrene of the left leg, leading to severe pain and deterioration in his functional status. He refused below knee amputation of the leg but received long-term antibiotics and was started on pain management with parenteral opioids.

While in hospital, patient underwent fiberoptic bronchoscopy with transbronchial biopsy. Visualization of right bronchial tree demonstrated extrinsic compression of the right lower lobe bronchus as well as distortion of anatomy likely due to radiation fibrosis. Transbronchial biopsy results were significant for small cell carcinoma (Fig. 3C) and the tumor cells were positive for synaptophysin, chromogranin and TTF-1, and were negative for p40 and leukocyte common antigen. The tumor cells showed a high proliferation activity of 95% by Ki-67. Due to poor performance status, he was not a candidate for systemic chemotherapy for SCLC. The patient and his wife decided to pursue symptom management to control his right leg pain and shortness of breath and went into hospice care.

## 3 Discussion

Patients treated for lung cancer are at risk of development of new malignant lesions in the future. Metachronous primary lung cancer needs to be differentiated from recurrent cancer; the differentiation is based on histopathology and a >4 year interval between the 2 diagnoses. The rate of developing a second primary lung cancer has been reported to be 1% to 2% per patient year after treatment for non-small cell lung cancer (NSCLC); and 6% per patient year after treatment for SCLC.^[3]^ A single center review of stage II and III lung cancer patients noted a cumulative risk of developing second primary lung cancer of 20% at 10 years post-surgery and 25% at 14 years post-surgery.^[4]^ This suggests a need for extended or lifelong surveillance. Patients who continue to smoke have an increased risk of developing a second lung cancer compared with those who do not.^[5]^ In a Surveillance Epidemiology and End Results analysis of patients with a diagnosis of lung cancer, the median 10-year risk of second primary lung cancer was 8.36%.^[6]^ The risk of a secondary cancer in survivors of SCLC is increased by 3.5-fold as compared to the general population. In patients who underwent radiation and continued to smoke, the risk was more than additive: the relative risk increased by 21-fold. The risk of a second lung cancer does not appear to plateau.^[7]^ A single center review of 5405 lung cancer patients showed that 3.5% had a concurrent malignancy. Of those patients with another malignancy, 63% had a metachronous malignancy and 37% had a synchronous malignancy. The most common other cancers in order were: colorectal, esophageal and thyroid. Notably, patients with metachronous malignancy had a better overall survival (OS) than synchronous malignancy with a median OS of 72.8 (range 12.2–391.0 months) and 12.9 (range 0.8–86.3 months) months, respectively (*P* < .001).^[8]^

Of the multiple primary lung cancer patients described in the literature, 2 had triple metachronous lung cancers^[9]^ (including our patient) and 4 had triple synchronous^[10–13]^ (Table 2). The only other patient with triple metachronous cancers described in the literature was a smoker who had 2 subsequent primary lung cancers.^[9]^ This patient initially developed SCLC followed by ADC and SCC within 4 years and underwent treatment with chemoradiation for SCLC and surgical resection for the subsequent cancers and then remained cancer-free.^[9]^ However, our patient developed SCLC after the 2 initial NSCLCs (ADC, SCC) over a span of 10 years. He continued to smoke and developed smoking-related complications (peripheral arterial disease) and was not a candidate for definitive treatment for subsequent cancers, leading to a worse outcome. The patients with synchronous and metachronous lung tumors had a mean age of 65 (range: 49–73 years), which places them below the average age of 70 at which lung cancer is diagnosed and had long history of smoking (≥50 pack-years).^[14]^

**Table 2:** Tumor identification and temporal details of patients with multiple lung cancers.

Age at first cancer/ Gender	First Cancer	Interval (months)	Second Cancer	Interval (months)	Third Cancer	Cancer occurrence	Smoking (pack-years)	Reference number
63/Male	ADC (acinar)	0	SCC	0	SCLC	S	55	10
49/Male	SCLC	0	ADC	0	Bronchial Carcinoid	S	60	11
74 (NA)	SCC	0	SCLC	0	ADC (acinar)	S	60	12
72/Male	SCC	0	ADC (mucinous)	0	ADC (acinar)	S	>45	13
73/Male	SCLC	29	ADC (acinar)	17	SCC	M	50	9
60/Male	ADC	108	SCC	6	SCLC	M	55	current case

Carcinogenic effects of cigarette smoke have been known for over 50 years, with the hazard ratio for developing lung cancer in smokers vs nonsmokers of 17.8 in women and 14.6 in men.^[15]^ The greatest predictor of lung cancer risk is pack-years of cigarettes smoked, with associated increase in risk with higher number of pack-years.^[15]^ The International Agency on Research on Cancer identified 72 measurable carcinogens in cigarette smoke, one such carcinogen is the polycyclic aromatic hydrocarbons (PAH).^[8]^ PAH-DNA adducts have been observed in lung tissues and p53 mutations in lung tumors may be similar to damage created by PAH metabolites in vitro.^[16]^ It has been hypothesized that a major pathway for cancer development is the smoke exposure leading to carcinogen-DNA adduct formation, which can cause mutations; unless repaired or removed by apoptosis mutations result in cancer.^[17]^ Polymorphisms in the genes coding for carcinogen metabolizing enzymes, DNA repair mechanisms, chromosomal fragility can affect the individuals cancer risk. Patients who quit smoking after cancer diagnosis have been shown to lower the risk of developing secondary cancers than those who continue to smoke.^[7]^ Smoking cessation continues to be a challenge despite the availability of multiple behavioral and pharmacologic interventions. Our patient had made multiple attempts to quit smoking but failed to maintain long-term success. A phase III trial of varenicline versus placebo has been shown to achieve abstinence from smoking with a rate of 29.7% in the varenicline group compared with 13.2% in the placebo group (OR, 2.83; 95% CI, 1.91–4.19; *P* < .001) at 24 weeks and 23% in the varenicline group compared with 10.3% in the placebo group (OR, 2.66; 95% CI, 1.72–4.11; *P* < .001) at 52 weeks.^[18]^ The second and third primary lung cancers in our patient were SCC and SCLC which are known to be associated with chronic smoking.

The duration of radiological monitoring is not clear for patients treated for initial lung cancer. Metachronous lung cancer patients undergoing surgery have been shown to have 5-year survival rates of 30% for all and 40% for stage I patients.^[2]^ Patients with lung cancers who continue to smoke should be placed on long-term, possibly life-long, radiological surveillance.

## 4 Conclusions

We have described a patient who developed 3 metachronous primary lung cancers. It highlights the adverse effects of continued smoking, and the importance of continued surveillance in such patients. The patients continued smoking and a history of radiation therapy substantially contributed to the development of multiple lung cancers, and together with his comorbid conditions (i.e., peripheral arterial disease) led to an extremely poor outcome. We glean from the literature that patients with multiple primary lung cancers are likely to be male with longer history of smoking and are diagnosed at a younger age. Long-term surveillance after initial treatment as well as persistent efforts at smoking cessation is important aspects of survivorship plans after treatment of lung cancer.

## Author contributions

D.D., B.B., J.M., L.C., and G.S. were involved in case formulation, writing manuscript and review of the literature. M.A.H., A.J. and R.G. were involved in providing histopathology of specimens including images and description. All authors were involved in reviewing and formatting of the manuscript.
